# Phase stability and dynamics of entangled polymer–nanoparticle composites

**DOI:** 10.1038/ncomms8198

**Published:** 2015-06-05

**Authors:** Rahul Mangal, Samanvaya Srivastava, Lynden A. Archer

**Affiliations:** 1 School of Chemical and Biomolecular Engineering, Cornell University, Ithaca, New York 14853, USA; 2 Institute for Molecular Engineering, The University of Chicago, Chicago, Illinois 60637, USA

## Abstract

Nanoparticle–polymer composites, or polymer–nanoparticle composites (PNCs), exhibit unusual mechanical and dynamical features when the particle size approaches the random coil dimensions of the host polymer. Here, we harness favourable enthalpic interactions between particle-tethered and free, host polymer chains to create model PNCs, in which spherical nanoparticles are uniformly dispersed in high molecular weight entangled polymers. Investigation of the mechanical properties of these model PNCs reveals that the nanoparticles have profound effects on the host polymer motions on all timescales. On short timescales, nanoparticles slow-down local dynamics of the host polymer segments and lower the glass transition temperature. On intermediate timescales, where polymer chain motion is typically constrained by entanglements with surrounding molecules, nanoparticles provide additional constraints, which lead to an early onset of entangled polymer dynamics. Finally, on long timescales, nanoparticles produce an apparent speeding up of relaxation of their polymer host.

## Abstract (web summary)

The lowering of polymer viscosity upon addition of small amounts of nanoparticles is counter-intuitive and has puzzled researchers. Here, Mangal *et al*. explain this intriguing phenomenon using a model polymer–nanocomposite system comprised of well-dispersed nanoparticles in an entangled polymer melt.

## Graphical abstract

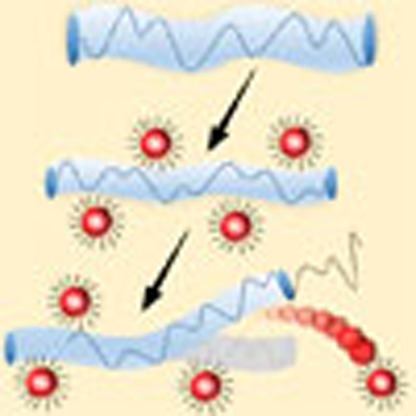

Addition of rigid nanoscale fillers to polymer melts is a well-known technique for augmenting various properties of polymeric materials, such as mechanical strength^1^,^2^,^3^, thermal stability^3^,^4^, barrier properties^5^,^6^, dimensional stability^7^ and wear resistance^8^. Remarkably, these particle-induced property enhancements are detected even at low volume fractions, where continuum analysis suggests minimal effect of the particles. As remarkable are the observations for polymer–nanoparticle composites (PNCs) in which the particle diameter (*D*) approaches the random coil radius (*R*_g_) of their host polymer. In these systems, even at low particle loadings NPs cause unusual viscosity reduction of the PNC relative to the particle-free host. This is in stark contrast to expectations based on the Einstein–Batchelor^9^,^10^ law for spherical particle suspensions, wherein addition of particles to a fluid always increases the viscosity of the fluid. In their seminal 1975 study, Malinskii and co-workers^11^,^12^ reported that addition of small amounts of unfunctionalized particulate fillers to high molar mass polymers produce an unexpected decrease in viscosity, followed by an increase at higher filler contents. These findings have since been extended to a variety of PNCs on the basis of unfunctionalized as well as polymer-functionalized NPs, including polystyrene^13^, magnetite^14^ and fullerene^14^,^15^ nanoparticles in polystyrene hosts, silsesquioxane–polymer composites^16^,^17^,^18^, and tethered silica–polymer composites^19^. The most recent studies have also established empirical NP and polymer size requirements to achieve the viscosity reductions, which have been investigated in detail by simulations^20^ and theory^21^,^22^; however, how and why NPs violate the Einstein–Batchelor viscosity law in polymers remains a puzzle.

It is understood that achieving good dispersion of particles in their host polymer is difficult, but a requirement for meaningful studies of PNCs. Malinskii and co-workers^11^,^12^ used a rapid quenching procedure, wherein particle/polymer solutions in a volatile co-solvent were quickly frozen, followed by lyophillization to remove the solvent. This approach is designed to trap the particles in the configurations they adopt in dilute solution, but is also known to produce large density variations and aging phenomena in the PNCs. More recent studies have employed steric stabilization with small molecules and polymers to weaken the characteristic, strong attractive surface forces between NPs; however, phase separation of even these hairy NPs has been reported to be the normal case in PNCs formulated using high molecular weight polymers^23^,^24^,^25^. Notwithstanding these challenges, multiple physical processes have been argued to contribute to the observed viscosity reductions. It has been proposed that NPs enhance the available free volume for polymer chains^11^,^12^ or act akin to molecular plasticizers and speed-up relaxation of the entangled host polymer^15^,^17^,^19^. Other lines of work suggest that NPs diffuse faster in high molar mass, entangled polymers and thereby lower the lifetime of entanglements in a manner analogous to constraint release^14^,^26^ produced by a lower molecular weight polymer in a polydisperse melt. The recent molecular dynamics study by Kalathi *et al*.^20^ and theoretical studies by Ganesan *et al*.^21^ and Wang *et al*.^22^ suggest polymer slip at a NP polymer interface as an additional factor that may explain how particles lower the polymer viscosity.

In this article we report on the phase behaviour and dynamics of model PNCs and highlight multiple new and subtle effects that shed light on the role NPs play on relaxation behaviours of entangled polymers. PNCs studied here are created by dispersing poly(ethylene glycol) (PEG)-grafted SiO_2_ nanoparticles in high molecular weight (*M*_w_) poly(methyl methacrylate) (PMMA) melts. By taking advantage of the slightly negative Flory–Huggins interaction parameter *χ* between tethered PEG and host PMMA molecules, we first show by means of small-angle X-ray scattering (SAXS) that it is possible to create PNCs with uniformly distributed NPs in entangled polymer hosts. Utilizing the structural simplicity of the materials, we study their relaxation dynamics over timescales spanning the complete range from fast monomer/segmental-scale motions to slow terminal relaxation. In particular, we find that NPs slow down polymer dynamics on all timescales, even at low particle concentrations. We also report that for well-entangled polymers, NPs play an active role in enforcing entanglement constraints on short timescales; their diffusion on longer timescales facilitates polymer relaxation and produces an apparent decrease in the terminal viscosity of the host polymer.

## Results

### Synthesis of model PNCs

Silica (SiO_2_) nanoparticles with average diameter *D*_avg_=10.8±0.3 nm (Supplementary Fig. 1) are densely grafted (Σ∼1.5–2 chains nm^−2^), with linear PEG of *M*_w_=450 and 2,000 g mol^−1^ using previously reported procedures^27^. Composites of these hairy NPs in linear PMMA and PEG of different molecular weights are created using chloroform as a co-solvent. Removal of the co-solvent followed by thermal annealing yields a library of PNCs with varying particle core fraction *φ*, host polymer chemistries and tethered and host polymer molar weights.

### Structural characterization of PNCs using SAXS

**Figure 1:**
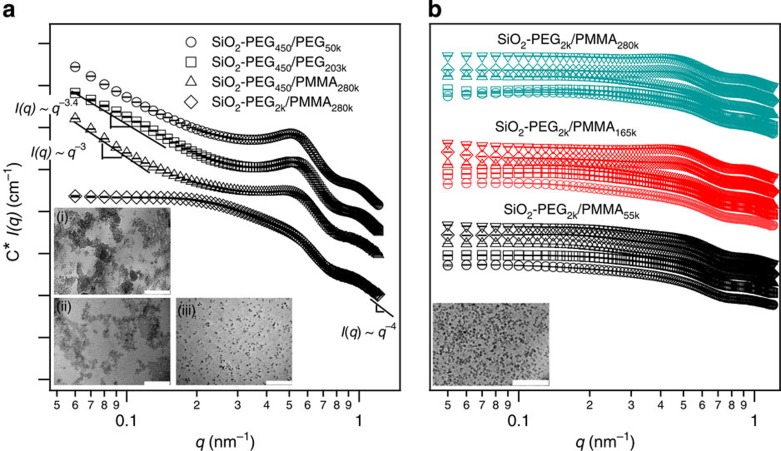
Phase stability of model nanoparticle-polymer composites. Scaled intensity C*I(q) versus wave vector *q* profile for **a**. SiO_2_–PEG_450_/PEG_50k_, SiO_2_–PEG_450_/PEG_203k_, SiO_2_–PEG_450_/PMMA_280k_ and SiO_2_–PEG_2k_/PMMA_280k_ at *φ*=2%. The insets (i), (ii) and (iii) are transmission electron micrographs of the SiO_2_–PEG_450_/PEG_50k_, SiO_2_–PEG_450_/PEG_203k_ and SiO_2_–PEG_2k_/PMMA_280k_ materials, respectively. (**b**) SiO_2_–PEG_2k_/PMMA_55k\165k\280k_ at *φ*=0.5% (circles), 1% (diamonds), 2% (upright triangles), 5% (inverted triangles) and 10% (squares). *I(q)* curves are displaced vertically for clarity of presentation. Scale bars in the TEM images represent the length of 200 nm. *C* is a scaling factor used to displace *I(q)* curves vertically in both the plots, for clarity of presentation. Errors in *I(q)* are the s.d.'s of the counts on the two-dimensional detector pixels with same *q* value, with the *I(q)* being the mean of all those values. The error bars shown in the figure are consistently smaller than the symbols.

Particle dispersion in these PNCs is investigated through SAXS measurements performed at Sector 12-ID-B of the Advanced Photon Source. In Fig. 1a, we report the scaled scattering intensity C*I(q) for PNCs comprising SiO_2_ particles tethered with PEG (*M*_w_=2 kg mol^−1^) suspended in high (*M*_w_=280 kg mol^−1^) atactic PMMA with polydispersity index (PDI)=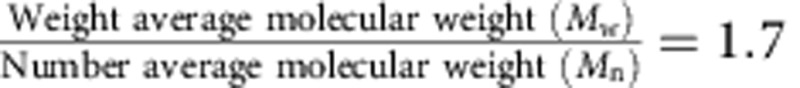) with *φ*∼2%. Here *C* is a scaling factor used to displace the curves vertically for better representation. Absolute values of *I(q)* for different samples are reported in Supplementary Fig. 2a. A long plateau in *I(q)* in the low wave vector (*q*) region for the SiO_2_–PEG_2K_/PMMA_280K_, a signature of well-dispersed particles in PNCs, is apparent in the figure. In contrast, for PNCs based on SiO_2_–PEG_450_ particles in either of the PEG (*M*_w_=50 kg mol^−1^; PDI=1.4 and 203 kg mol^−1^; PDI=1.14) or PMMA (*M*_w_=280 kg mol^−1^; PDI=1.7) hosts with *φ*∼2% loadings, *I(q)* increases as an approximate power-law function, *I*(*q*)∼*q*^−α^ in the low *q* range.

The strong dependence of *I(q)* on *q* at low wave vectors is a known characteristic of phase-separated or aggregated structures^24^ whose dimensions are beyond the length scales probed by the SAXS measurements. For the phase-separated PNCs, the power-law exponent *α* is between 3 and 4, indicating that NPs form compact aggregates and scatter similar to surface fractals^28^,^29^. From the transmission electron micrographs shown in the insets in Fig. 1a for (i) SiO_2_–PEG_450_/PEG_50k_, (ii) SiO_2_–PEG_450_/PEG_203k_ and (iii) SiO_2_–PEG_2k_/PMMA_280k_ materials at *φ*=2% and in the inset of Fig. 1b for SiO_2_–PEG_2k_/PMMA_280k_ at *φ*=5% NPs appear to exist as irregularly shaped islands in the former two cases and as a homogeneous dispersion in the latter ones. It is interesting to note that even in the phase-separated domains, NPs appear not to be aggregated, and a transmission electron microscopy (TEM) measurement that inadvertently sampled only one of these domains would conclude that the NPs are well dispersed, but with interparticle distances substantially lower than expected for a *φ*=2% suspension. Previous reports^23^,^30^ have shown how the degree of polymerization (*P*) of the host polymer to that of the tethered polymer (*N*) determines the degree of interpenetration and mixing between grafted layers and polymer melts. In particular, Srivastava *et al*.^23^ reported that PNCs with highly grafted NPs and characterized by *P*/*N*≥5 typically exist as phase-separated materials. For SiO_2_–PEG_450_/PEG_203k_, *P*/*N*≈450, it is therefore hardly a surprise that these NPs are phase-separated. In contrast, SiO_2_–PEG_2k_/PMMA_280k_ (*P*/*N*≈140) shows no evidence of particle phase separation, but SiO_2_–PEG_450_/PMMA_280k_ (*P*/*N*≈620) does. Blends of linear PMMA and PEG are widely known to be miscible because of favourable enthalpic monomeric interactions (*χ*<0; ref. 31). Although there is still no consensus regarding the exact value of *χ* for PMMA–PEG mixtures^32^,^33^,^34^, the attractive enthalpic interactions between the particle-tethered and host polymer can explain the better dispersion of SiO_2_–PEG_2k_ in PMMA hosts. Moreover, the enthalpic contribution to the overall PNC free energy is proportional to the number of overlapping segments 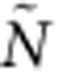 between the tethered PEG and host PMMA chains^35^,which would be lower for PEG_450_ and perhaps explains why SiO_2_–PEG_450_/PMMA_280k_ PNCs are phase-separated. In Fig. 1b we further report C*I(q) for a large number of SiO_2_–PEG_2k_/PMMA PNCs with varying core volume fractions and *P*/*N* values ranging from 140 to 27. Absolute values of *I(q)* for different samples are shown in Supplementary Fig. 2b–d. All of these systems manifest stable *I(q)* plateaus at low *q*, indicating that NPs are well dispersed. We also measured the second Virial coefficient *B*_2_ for our PNCs (*B*_2_,_PNC_) and for hard spheres (*B*_2,HS_), using a modified Zimm analysis, _lim*q*→0_1/*S*(*q*)=1+2*B*_2_*C* (ref. 36), where *S(q)*=*I*(*q*)/*I*(*q*=0) is the structure factor obtained from SAXS measurements. The results are shown in Supplementary Table 1. It is evident that *B*_2,PNC_ is always positive and consistently larger than *B*_2,HS_, implying that the tethered PEG chains are highly stretched out on the particle surface, consistent with expectations for polymer-grafted particles in a good solvent^36^,^37^. Our observations imply that the stability of the particles originates from the strong steric stabilization provided by the extended brush of tethered PEG chains, interacting enthalpically with the host PMMA chains. This finding agrees with previous reports, which demonstrate enhanced phase stability of polymer-grafted nanoparticles in host polymers that interact favourably with the grafted chains^38^.

### Effect of nanoparticles on the dynamics of host polymer chains

**Figure 2:**
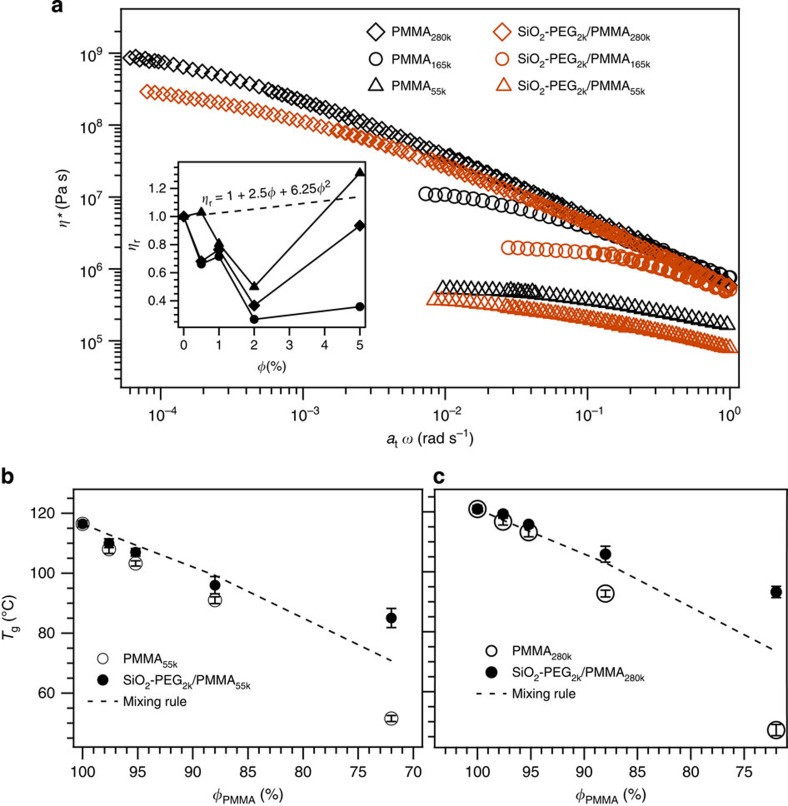
Effect of NPs on dynamic viscosity and glass transition temperature of polymers. (**a**) Plot of complex viscosity (η*) versus shifted frequency (*a*_t_ ω) for neat PMMA and for PNCs SiO_2_–PEG_2k_/PMMA_55k_, SiO_2_–PEG_2k_/PMMA_165k_ and SiO_2_–PEG_2k_/PMMA_280k_ with *φ* =2% at *T* =190 °C. Similar behaviour was observed in steady shear measurements (Supplementary Fig. 4). Inset shows the relative viscosity (*η*_r_=*η*_PNC_/*η*_neat_) for SiO_2_–PEG_2k_/PMMA_55k_ (triangles) from steady shear measurements at 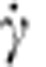
∼0.02 s^−1^ at *T*=180 °C, for SiO_2_–PEG_2k_/PMMA_165k_ (circles) at 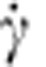
∼0.02 s^−1^ at *T*=190 °C and for SiO_2_–PEG_2k_/PMMA_280k_ (diamonds) at 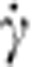
∼0.001 s^−1^ at *T*=170 °C. The dashed line is the predicted relative viscosity from the Einstein–Batchelor equation^9^,^10^. Error bars smaller than symbol size are not shown. (**b**,**c**) *T*_g_ values obtained using DSC for SiO_2_–PEG_2k_/PMMA_55k_ and SiO_2_–PEG_2k_/PMMA_280k_, respectively. Closed symbols correspond to PNCs and open symbols correspond to corresponding particle-free neat blends. Dashed lines represent the data computed using the mixing rule predicted by the Fox equation 
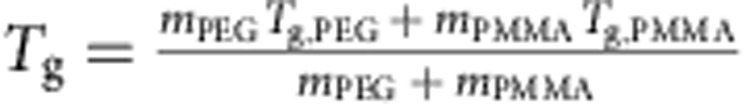
. DSC measurements are performed at different scan rates (2, 5, 10, 15 and 20 K min^−1^) and obtained *T*_g_ values showed no dependence on scan rates (Supplementary Figs 5 and 6). Results for SiO_2_–PEG_2k_/PMMA_165k_ are shown in Supplementary Fig. 7.

The phase stability of SiO_2_–PEG_2k_ nanoparticles in entangled PMMA melts allows us to use these materials as structure-less, model PNCs for studying how NPs affect their host polymer dynamics. In Fig. 2a, we compare the complex viscosity of PMMA melts and SiO_2_–PEG_2k_/PMMA_55k\165k\280k_ PNCs with *φ*=2%. It is apparent that NPs lower the polymer viscosity in all PNCs studied, particularly at low rates, which is opposite to large increases in viscosity, observed in PNCs with phase-separated particles^39^. It also defies the Einstein–Batchelor prediction^9^,^10^ for the viscosity increase in suspensions of spherical particles. Similar behaviour is observed up to *φ*∼5% (inset to Fig. 2a); however, the polymer viscosity is seen to increase with particle loading at higher *φ* values. Our results are evidently consistent with earlier reports that addition of NPs to high molar mass polymers at low concentrations lowers the viscosity^13^,^14^,^15^,^16^,^17^,^18^,^19^. In this case, however, NPs lower the host polymer's viscosity irrespective of the number of entanglements per chain Z≈*M*_w_/*M*_e_, where *M*_e_ is the entanglement molecular weight, which varies from ∼5 (PMMA_55k_) to 28 (PMMA_280k_); *M*_e,PMMA_, is 10 kg mol^−1^ (ref. 40).

The glass transition temperature (*T*_g_) for the SiO_2_–PEG_2k_/PMMA composites and corresponding particle-free PMMA/PEG blends are measured through differential scanning calorimetry (DSC; Supplementary Figs 5 and 6). As shown in Fig. 2b,c, a decreasing trend of *T*_g_ with decreasing PMMA volume fraction (*φ*_PMMA_) for both the SiO_2_–PEG_2k_/PMMA PNCs and the PMMA/PEG blends is observed. Decreasing *T*_g_ with decreasing glassy polymer content in a homogeneous mixture is the expected response to a plasticizer, which increases free volume of the polymer. The dashed lines are the values predicted by the Fox relation^41^ for *T*_g_ of plasticized polymers.

It is clear that free PEG has an appreciably larger plasticizing effect than expected from the Fox relation. Such negative deviations in *T*_g_ of PEG/PMMA mixtures, relative to the Fox relation, have been reported previously and argued to be a consequence of the segmental-scale attraction of PEG and PMMA^42^. At the same time, a more pronounced decrease observed for free PEG chains relative to tethered chains can be understood both in terms of the lower number of chain ends^43^ and lower mobility near SiO_2_ particle surface^44^ for the latter.

**Figure 3:**
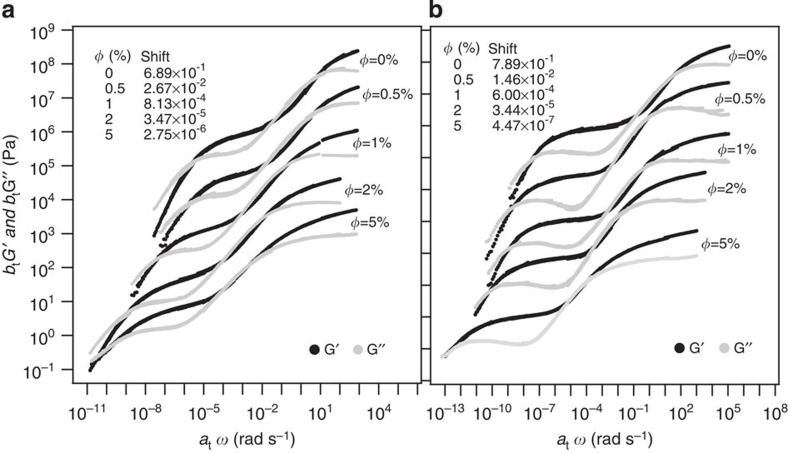
Time–temperature superposition master curves showing the effect of NPs on polymer dynamics. Master curves (dynamic moduli versus shifted frequency) obtained from TTS of oscillatory shear rheology measurements for: (**a**) SiO_2_–PEG_2k_/PMMA_55k_ and (**b**) SiO_2_–PEG_2k_/PMMA_280k_. Master curves for different *φ* values have been shifted vertically for clarity of presentation, shift factor for which are mentioned in the figure legend. Frquency sweep measurements at temperatures *T*>*T*_g_ are shifted with respect to the material's respective *T*_g_'s, to create each master curve. Typical shift factors employed in obtaining the individual master curves are shown in Supplementary Fig. 10. Error bars are smaller than symbol sizes and are not shown.

The effect of nanoparticles on the dynamics of PMMA was studied in detail using frequency-dependent oscillatory shear measurements of pure PMMA and SiO_2_–PEG_2k_/PMMA PNCs at multiple discrete temperatures, *T*>*T*_g_. The time–temperature superposition procedure was used to create dynamic maps or master curves shifted (Supplementary Fig. 8) at a fixed temperature distance with respect to the measured *T*_g_ values for respective material. This shifting procedure eliminates the trivial plasticization effect caused by the increase in free volume resulting from addition of nanoparticles. As illustrated in Fig. 3a,b, these measurements provide complete information on how the NPs affect the host polymer dynamics on timescales that span the entire range from segmental-scale motions to terminal/reptation relaxation. It is also evident from both figures that with successive addition of NPs, the frequency-dependent dynamic moduli for SiO_2_–PEG_2k_/PMMA_55k_ and SiO_2_–PEG_2k_/PMMA_280k_ shift to the left, that is, towards lower frequency.

**Figure 4:**
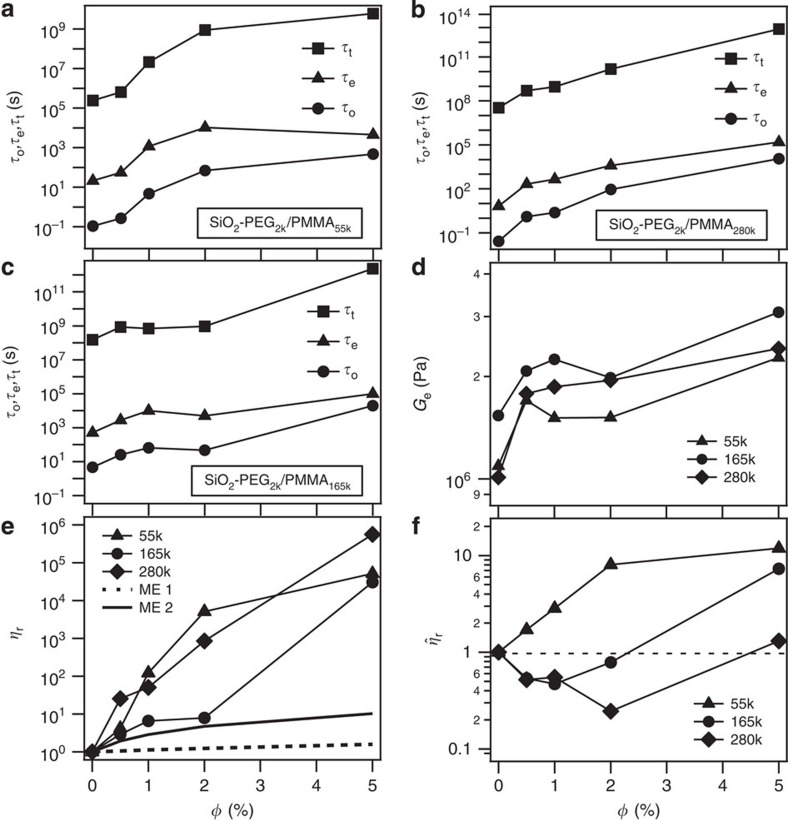
Effect of NPs on relaxation times, rubbery plateau modulus and relative viscosity of polymers. Effect of NP content in SiO_2_–PEG/PMMA PNCs on (**a**), (**b**), (**c**) the three relaxation times (*τ*_o_,*τ*_e_,*τ*_t_) for: SiO_2_–PEG_2k_/PMMA_55k_; SiO_2_–PEG_2k_/PMMA_165k_; and SiO_2_–PEG_2k_/PMMA_280k_ respectively. The relaxation times are obtained from the G' and G” crossover (Supplementary Fig. 8) at a fixed temperature difference from *T*_g_. (**d**) Plateau modulus (*G*_e_) obtained using Van Gurp analysis (Supplementary Fig. 9), as a function of particle concentration. (**e**) Relative viscosity *η*_r_=*η*_PNC_/*η*_neat_, where *η*∼*G*_e_*τ*_t_. The lines are predictions based on the modified Einstein (ME) expression 
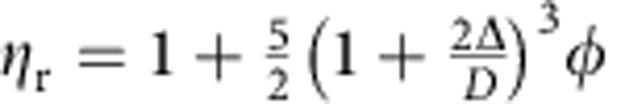
 (ref. 46), which accounts for a polymer brush of height; ME 1: Δ=2*R*_g_ (dashed line) or ME 2: Δ=fully stretched length (solid line). (**f**) Modified relative viscosity 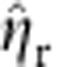.

The shift can be quantified in terms of the three fundamental timescales for entangled polymers^45^—the segmental relaxation time *τ*_o_, entanglement relaxation time *τ*_e_ and the terminal relaxation or reptation time *τ*_t_. In Fig. 4a–c we report the respective timescales^45^ obtained for all three PNCs (see Supplementary Fig. 8 for detailed analysis). It is apparent that addition of SiO_2_–PEG_2k_ NPs to PMMA slows down polymer relaxation on all timescales. The terminal Newtonian viscosity *η*_o_ of an entangled polymer can be estimated as *η*_o_*≈G*_e_*τ*_t_ where *G*_e_ is the shear modulus at the rubbery plateau. The procedure reported in ref. 46 was used to determine *G*_e_ (Supplementary Fig. 9). As shown in Fig. 4d, it can be seen that *G*_e_ increases continuously on nanoparticle addition; however, the changes are more modest than those seen for*τ*_t_. In Fig. 4e we plot the relative viscosity *η*_r_=*η*_PNC_/*η*_neat_ for these PNCs. Comparison of the results with those in the inset of Fig. 2a shows that once the plasticization effect is taken into account by comparing viscosities at a fixed temperature distance relative to *T*_g_ of the PNC, the conventional suspension result—namely that addition of particles always increases the suspension viscosity—is qualitatively recovered. More careful inspection of Fig. 4e indicates that the observed increase in *η*_r_ at modest amounts of NPs is far larger than what is normally achieved with hydrodynamic effects alone. To make this point more concrete, we computed *η*_r_ based on a modified Einstein dilute suspension expression^47^ 
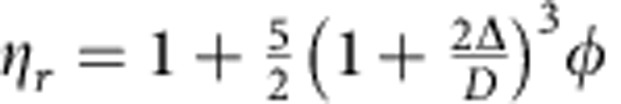
 to account for the contribution of tethered PEG-chain height (Δ) in nanoparticles' effective volume. At first, we evaluated *η*_r_ assuming tethered PEG chains to form a collapsed brush of height 2*R*_g_ (ref. 48; ∼1.7 nm for PEG_2k_), as shown by the dashed line. However, it is known from previous measurements^44^ that because of the high grafting density of tethered chains, this height can be substantially larger. Hence, we also evaluated *η*_r_ assuming the polymer brush to be comprising fully stretched out PEG chains with Δ∼N*b (∼16 nm), as shown in solid line, here *N* is the number of kuhn monomers in entire chain length and *b* is the length of a polymer chain segment. It is apparent that in both extreme limits, we are unable to reproduce the magnitude of the experimentally observed enhancements in viscosity.

In entangled polymer melts, relaxation is understood to proceed in a hierarchical manner beginning with fast, local segmental-scale processes on timescale *τ*_o_, which affect dynamics on longer timescales: *τ*_e_=(*a*_e_/*b*)^4^*τ*_o_ and *τ*_t_=3*Z*^3^*τ*_e_, where *a*_e_ is the entanglement tube diameter, *b* the length of a chain segment and *Z*=*M*/*M*_e_ is the average number of entanglements each chain makes with its neighbours. The large increases in *η*_r_ with increasing *φ* therefore to some extent reflect the strong increase in *τ*_o_ apparent in Fig. 4a–c. This effect can be taken into account by defining a modified relative viscosity 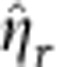 
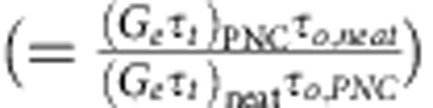
, in which *τ*_t_ is normalized using the measured *τ*_o_ values.

From Fig. 4f, we understand that, while 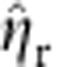 increases progressively with increasing particle content for PNCs on the basis of the 55k PMMA host, for materials based on the 165k and 280k PMMA host polymers, 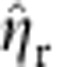 decreases at low *φ*, but increases at high *φ*. This effect of NPs on 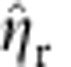 is in qualitative accord with earlier reports, but would obviously only be visible in experiments in which the NPs do not also slow down segmental-scale dynamics. In addition, in the present case, the apparent reduction in viscosity is isolated from any NP plasticization or other NP effects on segmental-scale dynamics, implying that neither of these two arguments advanced in the early literature can be the source of the behaviour.

**Figure 5:**
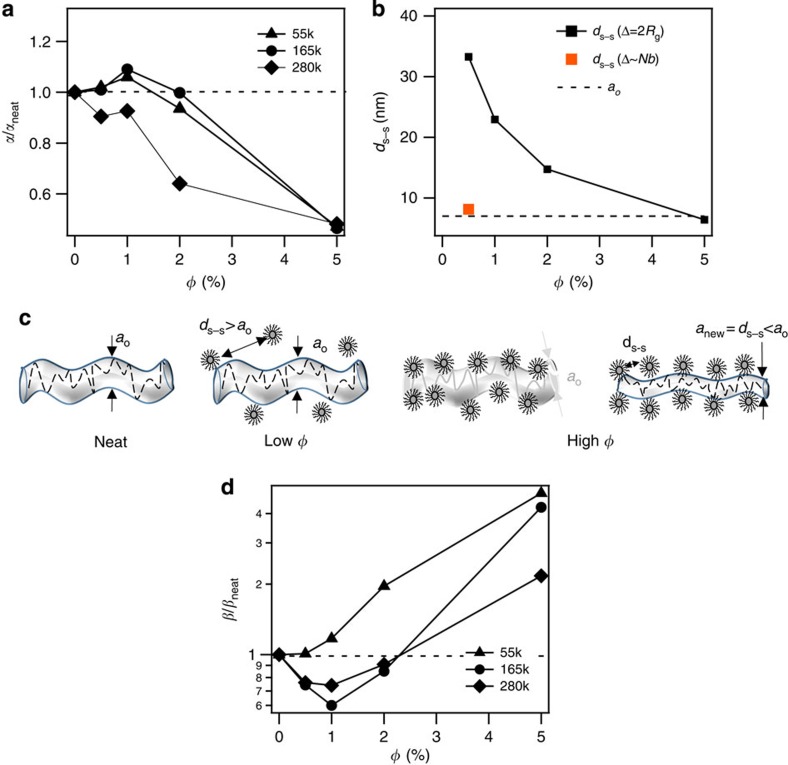
Effect of NPs on entanglement tube diameter and effective number of entanglements of host polymer chains. (**a**) The effect of NP volume fraction on *α*≡(*τ*_e_/*τ*_0_)^1/4^=*a*_e_/*b* for PNCs with respect to the particle-free melt. (**b**) Surface-to-surface distance (*d*_*s–s*_=*d*_*p–p*_−(2Δ+*D*)), for Δ=2*R*_g_ and Δ∼*Nb*, as a function of particle content. The dashed horizontal line corresponds to the tube diamater (*a*_o_) of the host polymer. (**c**) Schematic of the mechanism through which hairy NPs exert entanglement-like constraints on polymer chains in a PNC. (**d**) *β*≡(*τ*_t_/*τ*_e_)^1/3^=3^1/3^*Z* versus NP volume fraction for PNCs with respect to particle-free polymer melts.

More insights about the environment in which polymer chains relax in PNCs can be obtained by analysing the effect of NPs on entanglement dynamics of their host polymer. The dimensionless group *α*≡(*τ*_e_/*τ*_0_)^1/4^=*a*_e_/*b* allows this effect to be isolated. In Fig. 5a we report *α* for PNCs relative to the respective particle-free, neat PMMA (*α*_neat_). For PMMA_55k_ and PMMA_165k_, *α* is virtually unchanged up to *φ*≈2%, where after it falls sharply below the value of the neat PMMA melt indicating that the host polymer feels an effectively smaller tube diameter in the PNC. For PMMA_280k_, the most entangled polymer studied, *α* is always lower than *α*_neat_ (although moderately at low *φ*) meaning that the PMMA chains in PNC are in a more constricted tube than in pure melts at all *φ*. Through the cartoon in Fig. 5c we intend to help one visualize the role NPs might play in constraining their host polymer chains in tighter tubes. We also estimated the interparticle distance *d*_*p–p*_ in a well-dispersed PNC on the bass of spherical packing using the formula 
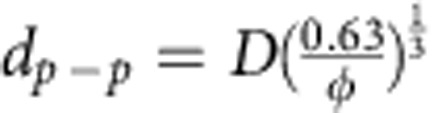
 and calculated surface-to-surface distance *d*_*s*–*s*_=*d*_*p*–*p*_−(2Δ+*D*). To obtain an estimate of the order of magnitude for *d*_*s–s*_, we again assumed the brush height as Δ≈2*R*_g._ In Fig. 5b we compare the calculated *d*_*s–s*_ obtained at various *φ* with the equilibrium tube diameter *a*_o_(=*bN*_e_), where *N*_e_ is the number of Kuhn monomers in *M*_e_. For *φ*<5%, as *d*_*s*–*s*_>*a*_*o*_, particles are sufficiently far apart and hence they are unable to confine the host polymer. However, at *φ*=5%, *d*_*s*−*s*_<*a*_*o*_, suggesting a tube-like particle-induced confinement effect on length scales of *d*_*s–s*_ is possible, which is consistent with our observations. For the case of a fully extended brush of tethered chains, we find that *d*_*s*–*s*_>*a*_o_ only for *φ*=0.5%, where after the surface-to-surface distance between particles is lower than the equilibrium tube diameter. Our results therefore show that even at low concentrations NPs in a PNC may provide additional, tube-like constraints on the motions of their entangled polymeric hosts. This conclusion is consistent with recent predictions from computer simulations^49^,^50^ and theory^51^. It also explains the earlier onset of in-tube/reptation motion of the host polymer chains observed in PNCs.

From the corresponding results for *β*≡(*τ*_t_/*τ*_e_)^1/3^=3^1/3^*Z* reported in Fig. 5d it is observed that for the two more entangled polymers there is a clear decrease in the effective number of entanglements *β* at low *φ* followed by a rise at high *φ*, whereas for PMMA_55k_ the least entangled polymer *β* monotonically increases with *φ*. That *G*_e_ that remains essentially unchanged over the same range of *φ* suggests that the observed reduction in *Z* for more entangled PMMA is not due to increased *M*_e_ (*=*
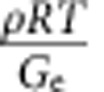
, where *ρ* is polymer density and *R* is the universal gas constant), but rather reflects a reduction in the number of effective entanglements experienced by the host chains at the time of tube escape.

This ‘constraint release' phenomenon has previously been theorized by Mackay and co-workers^14^ as responsible for the observed lowering of an entangled polymer's viscosity by NPs. In that case it was argued to originate from the fast diffusion of NPs in an entangled polymer host. Our results offer a complementary, although perhaps more straightforward explanation—because the NPs participate explicitly in the tube constraints the host polymer experiences on short timescales, their diffusion on longer timescales can reduce entanglements and perforate the tube in a manner analogous to what has been reported in bi-disperse polymer blends. In PNCs where the particle size (*D*∼10 nm) is only slightly larger than the host polymer entanglement mesh size (*a*_o_*∼* 7 nm in our case), particle motion and host-chain entanglement dynamics are expected to be coupled^52^ with the particle's escape time falling somewhere between *τ*_e_ and *τ*_t_ (ref. 50). The dissimilar effect of NPs on the terminal relaxation of weakly and well-entangled PMMA can therefore be understood in terms of competition between the rate of particle diffusion, which destroys the tube, and reptation relaxation of the polymer chains in their tube. In particular, we conjecture that for the more entangled PMMA hosts nanoparticle diffusion occurs on a timescale that causes tube reconstruction before the host is able to escape by reptation, which releases entanglements^26^,^53^. For the less entangled host, tube reconstruction by particle diffusion occurs at a rate comparable to or slower than tube escape by reptation.

### Role of tethered chains on the dynamics of host polymer chains

**Figure 6:**
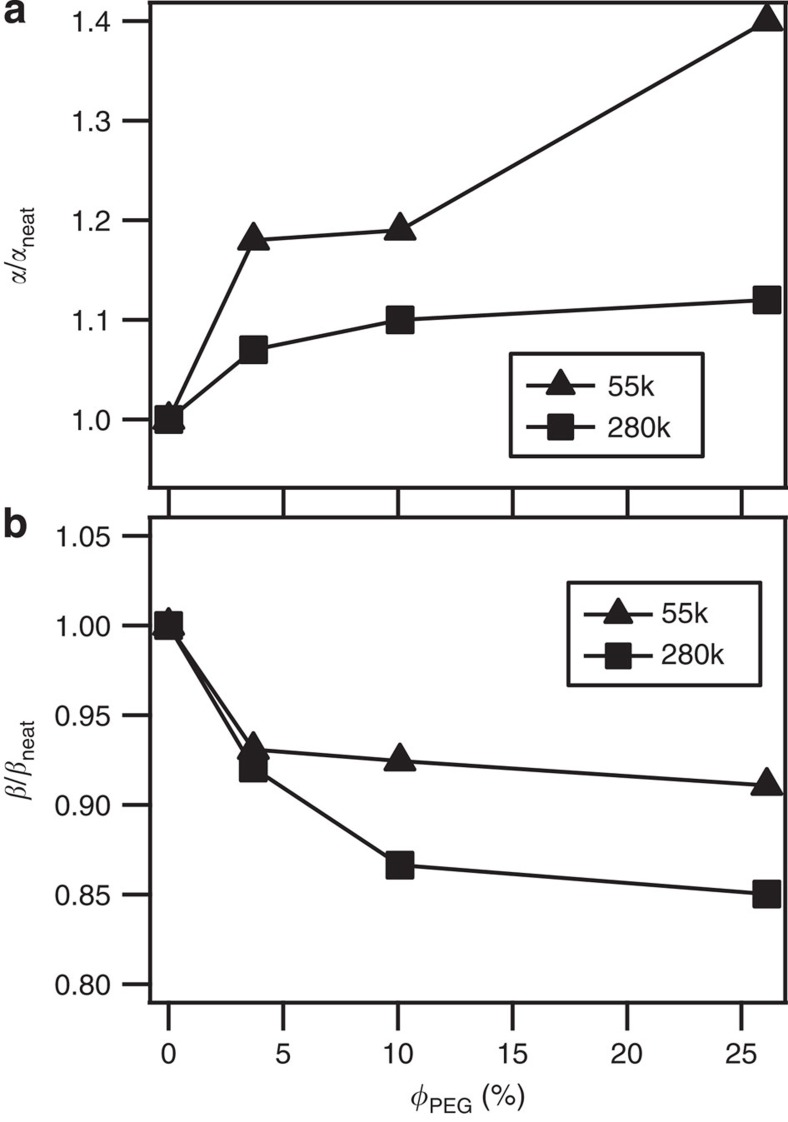
Effect of free PEG_2k_ chains on entanglement tube diameter and effective number of entanglements of host polymer chains. (**a**) *α*≡(*τ*_e_/*τ*_0_)^1/4^=*a*_e_/*b* and (**b**) *β*≡(*τ*_t_/*τ*_e_)^1/3^ =3^1/3^*Z* as functions of PEG_2k_ content for particle-free PEG_2k_/PMMA blends with respect to the respective pure PMMA materials.

We close by considering how tethered PEG chains might potentially affect the observations discussed here. From Fig. 6a,b we report the effect of PEG volume fraction (*φ*_PEG_) on *α* and *β* for linear PEG_2k_/PMMA_55\280k_ blends. By focusing on *φ*_PEG_ close to those of the corresponding tethered PEG loadings in the SiO_2_–PEG_2k_/PMMA PNCs, it is possible to characterize the effect of PEG on PMMA dynamics under conditions similar to those in the PNCs. The results clearly show that irrespective of the PMMA molecular weight, *α* increases and *β* decreases progressively with increasing *φ*_PEG_, suggesting that free PEG chains dilate the PMMA entanglement network. That the effect is qualitatively unchanged for the weak- and well-entangled PMMA molecules, suggests that the observed effect of NPs is a characteristic of the particles, as opposed to the chains tethered to their surfaces alone.

## Discussion

Our findings provide important new insights about how well-dispersed surface-functionalized nanoparticles influence the dynamics of polymers in nanocomposite materials. We show that favourable enthalpic interactions between the grafted and host chains can be used to overcome the tendency of nanoparticles to phase-separate in high molecular weight polymer hosts. In addition, we report for the first time that at a fixed temperature difference from *T*_g_, NPs slow down polymer relaxation on all timescales, including the segmental relaxation time, and over a broad range of particle concentrations. By studying the effect of NPs on the Rouse time for entanglement segments, we find that even at low particle concentrations, polymer-functionalized NPs provide additional tube-like constraints on chain relaxation that leads to an earlier onset of reptation relaxation. On long timescales, particle motions appear to degrade the tube surroundings of their host chains, which accelerates tube escape by a process analogous to constraint release in mixtures of entangled polymers with widely differing molecular weights. In well-entangled materials these processes lead to an apparent lowering of the viscosity of the host polymer at low NP concentrations. However, the effect would only be directly observable in experiments where the particles do not also slow down the host polymer's fast, segmental-scale motions.

## Methods

### Preparation of PNCs

Silane-functionalized polyethylene glycol chains (PEG, *M*_w,teth_∼450 g mol^−1^, PDI: 1.2, supplied by Gelest Inc.) were tethered to SiO_2_ nanoparticles of diameter ∼10 nm (LUDOX SM-30; supplied by Sigma Aldrich) to prepare SiO_2_–PEG_450_ particles. To synthesize SiO_2_–PEG_2k_ particles, silica particles were first functionalized with sulfonic acid using 3-(trihydroxysilyl)-1-propanesulfonic acid (supplied by Gelest Inc.). Later, amine-terminated PEG (*M*_w,teth_∼2 kg mol^−1^, PDI: 1.3, supplied by Polymer Source Inc.) was added in stoichiometric ratios. Repeated precipitation using a solvent (ethanol/chloroform) and a nonsolvent (n-hexane) was performed to completely remove the untethered polymer chains in both the cases. Thermogravimetric analysis (TGA), carried out on the TA Instruments TGA Q500 for successive repeated centrifuge cycles, showed no changes in solid content by the fifth centrifuge cycle, indicating negligible amount of the unattached material content and suggested the grafting density of ∼1.5–2 chains nm^−2^. Composites of the nanoparticles were prepared by mixing appropriate amounts of nanoparticles with host polymer PEG/PMMA (supplied by Polymer Source Inc) in a common solvent (chloroform) followed by removal of the solvent by heating for 12 h in convection oven at 70 °C, followed by heating for at least 12 h under vacuum at 50 °C for SiO_2_–PEG/PEG suspensions and at 130 °C under vacuum for SiO_2_–PEG/PMMA suspensions. For control measurements, particle-free neat PEG2k/PMMA blends, corresponding to the suspensions, were also synthesized. PEG_2k_ and PMMA were mixed in relative amounts similar to their respective suspensions again in a common solvent followed by removal of the solvent by heating for at least 12 h at 130 °C under vacuum.

### Determination of the glass transition temperature

DSC measurements were carried out at temperature ramp rates of 2, 5, 10, 15 and 20 K min^−1^ in a nitrogen environment using a TA Instruments DSC Q2000. *T*_g_ values were obtained from the infection point in heat capacity versus temperature plot as shown in Supplementary Fig. 5.

### SAXS measurements

SAXS measurements were performed at Sector 12-ID-B of the Advanced Photon Source. Measurements at 12-ID-B were carried out using Pilatus 2M detector, sound-bounce monochromator and a beam energy range of 7.9–14 keV. Measurements for SiO_2_–PEG/PEG composites were carried out at 70 °C and for SiO_2_–PEG/MMA composites at 130 °C. The scattering of NPs in the PNCs is obtained by subtracting the particle-free polymer-scattering, obtained using the same procedure. Errors in scattered intensities *I(q)* are the s.d.'s of the counts on the two-dimensional detector pixels with same wave vector *q*, with the *I(q)* being the mean of all those values.

### Rheology measurements

Steady shear rheology measurements were performed at 190 °C using a strain-controlled ARES–LS rheometer (Rheometric Scientific) outfitted with a 4-mm diameter cone and plate fixture. Measurements were performed at shear rates (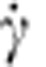) in the range 10^−4^ s^−1^≤
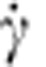
≤10^−1^ s^−1^. Oscillatory rheology measurements were performed at different temperatures (*T*), again using the same Rheometrics ARES rheometer outfitted with a 3-mm diameter parallel plate fixture. Measurements were performed in the linear viscoelastic regime at frequencies (*ω*) in the range 10^−1^ s^−1^≤*ω*≤10^2^ s^−1^. Thus, obtained frequency sweep curves were shifted horizontally and vertically, considering the data at *T*_g_ as reference, to obtain Time Temperature Superposition master curves. A representative master curve and typical shift factors are shown in Supplementary Figs 8 and 10, respectively. Plateau modulus (*G*_e_) values were obtained utilizing Van Gurp–Palmen plot^46^ in which phase angle *δ* (=tan^−1^(*G′′/G′*)) is plotted against |G*|. The analysis predicts that plateau modulus is approximately equal to the minimum value of |G*| (i.e. G_e_∼|G*|_*δ* min_).

## Additional information

**How to cite this article:** Mangal, R. *et al*. Phase stability and dynamics of entangled polymer–nanoparticle composites. *Nat. Commun*. 6:7198 doi: 10.1038/ncomms8198 (2015).

## Supplementary Material

Supplementary Information Supplementary Figures 1-10 and Supplementary Table 1
