# Supplementary material for: Phase stability and dynamics of entangled polymer–nanoparticle composites
Source: Nat Commun. 2015 Jun 5;6:7198. doi: 10.1038/ncomms8198 (PMC4468852; doi:10.1038/ncomms8198)
Supplement: Supplementary Information — Supplementary Figures 1-10 and Supplementary Table 1 [file ncomms8198-s1.pdf]

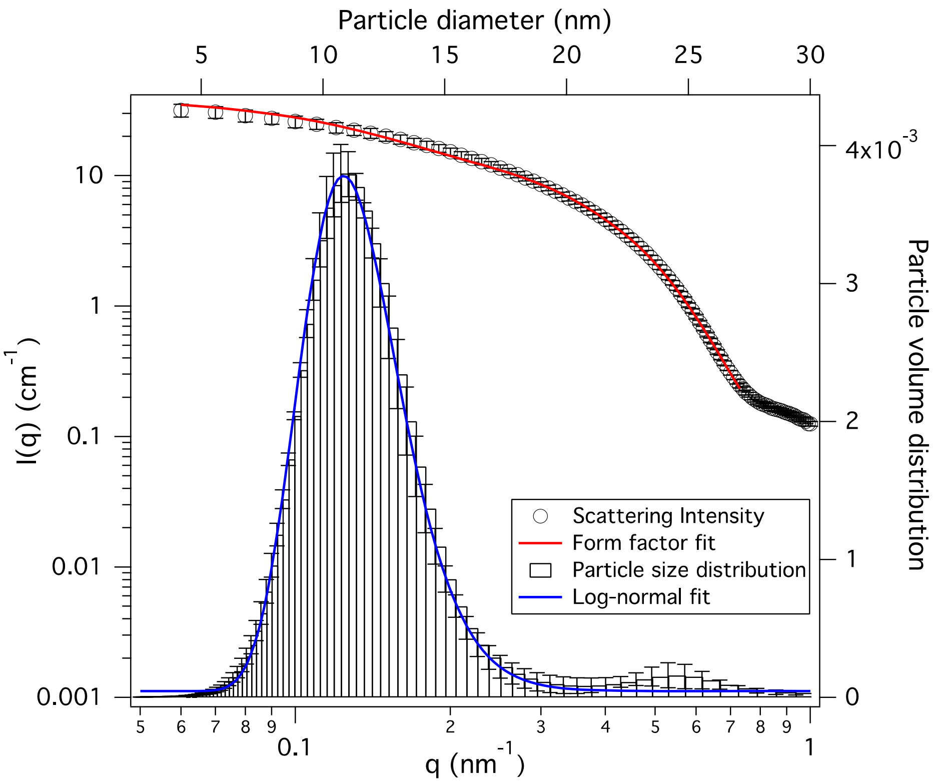

Supplementary figure 1. Particle size distribution obtained from SAXS measurements for 0.95 % w/w sulfonic acid functionalized silica nanoparticles in water.

The fitting of the scattering intensity was carried out using the size distribution tool of the SAXS data-modeling package IRENA (http://usaxs.xray.aps.anl.gov/staff/ilavsky/irena.html).

The resultant particle size distribution was found to be well described by a log normal distribution of the form $A\exp\left(-\frac{D/D_{avg}}{\sqrt{2}\sigma}\right)^2$, as shown in the blue line in the figure. Here A, D_{avg} and σ are an arbitrary pre-factor, the average diameter and the standard deviation in the particle diameter, respectively. The average particle diameter and the standard deviation in the particle diameters are found to be 10.8 ± 0.008 nm and 0.3 ± 0.001, respectively, as determined from the fit.

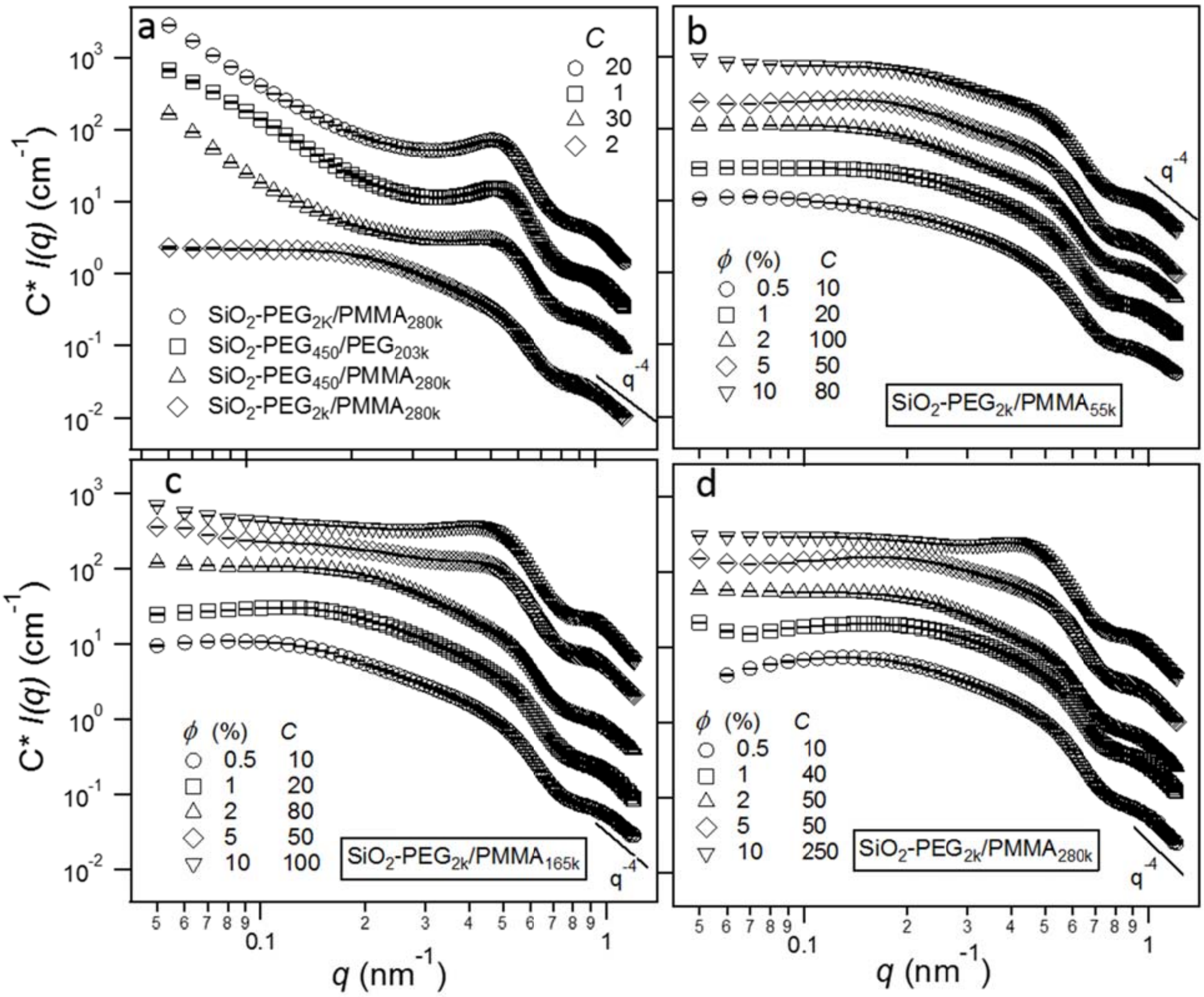

Supplementary figure 2. a. Comparison of scaled Intensity C**I(q)* vs wave vector *q* profile for PNCs with SiO_2-PEG_{450} and SiO_2-PEG_{2k} particles. Intensity *I(q)* vs wave vector *q* profile for **b**.SiO_2-PEG_{2k}/$PMMA_{65k}$ **c.**SiO_2-PEG_{2k}/$PMMA_{165k}$ and **d.**SiO_2-PEG_{2k}/$PMMA_{280k}$ at different particle concentrations (ϕ). Here *C* is the scaling factor used to shift the curves vertically in all the plots for the sake of clarity of presentation. Values of *C* have been mentioned as legends in the figures. Errors in I(q) are the standard deviations of the counts on the two-dimensional detector pixels with same q value, with the I(q) being the mean of all those values. The error bars shown in the figure are consistently smaller than the symbols.

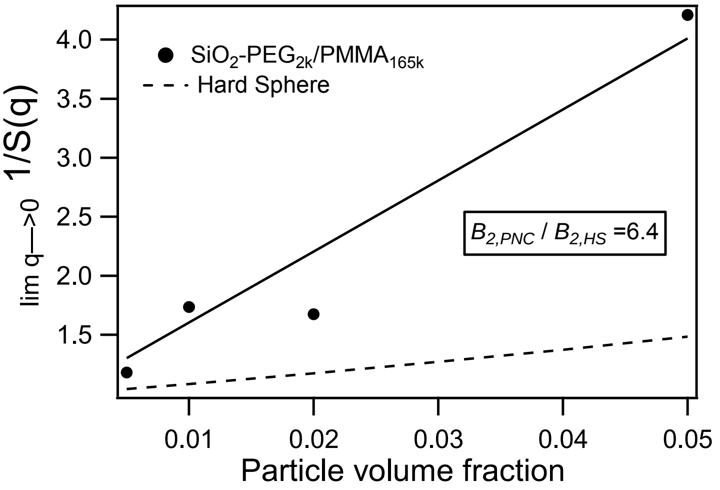

Supplementary figure 3. $\lim_{q\to 0} 1/S(q) = 1 + 2B_2C$ for SiO_2-PEG_{2k}/$PMMA_{165k}$ and hard spheres. Similar results were obtained for other PNCs and the extracted second Virial coefficient values for PNCs ($B_{2,PNC}$) are shown in Supplementary table 1 in comparison with $B_{2,HS}$.

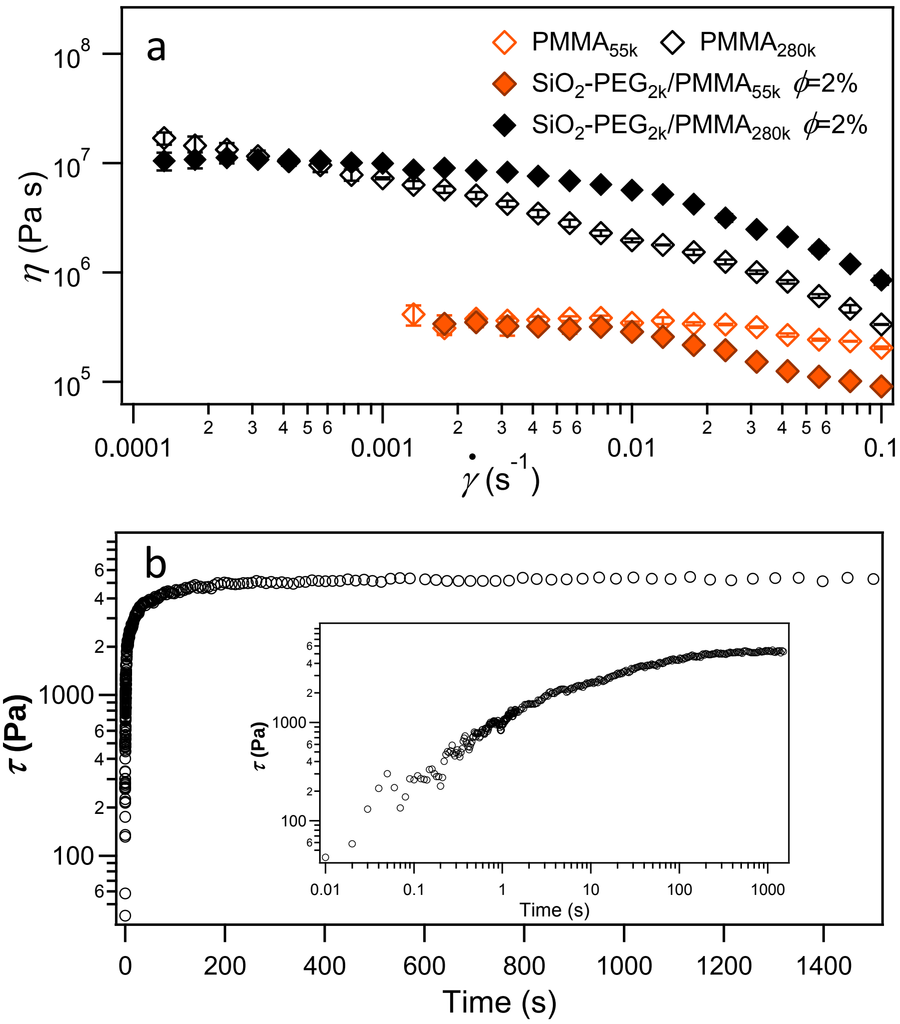

Supplementary figure 4. a. Results of Steady shear experiments measured at 190°C. Experiments were perfomed at Ares Rheometer, at a fixed shear rate $\dot{\gamma} = 0.0133\,\text{sec}^{-1}$. Sufficient waiting time was allowed for shear stress value to attain a steady state value resulting in plateau region as shown. Average shear stress value in the plateau region was considered to compute the viscosity $(\eta) = \frac{\text{Shear Stress }(\tau)}{\text{Shear Rate }(\dot{\gamma})}$ at that shear rate and to generate the flow curve. Error bars represent one standard deviation. **b**. Shear Stress vs time for neat $PMMA_{55k}$ at 190°C and at $\dot{\gamma} = 0.0133\,\text{sec}^{-1}$. Inset represents the same data in a log-log scale at same shear rate. Error bars smaller than symbol size are not shown.

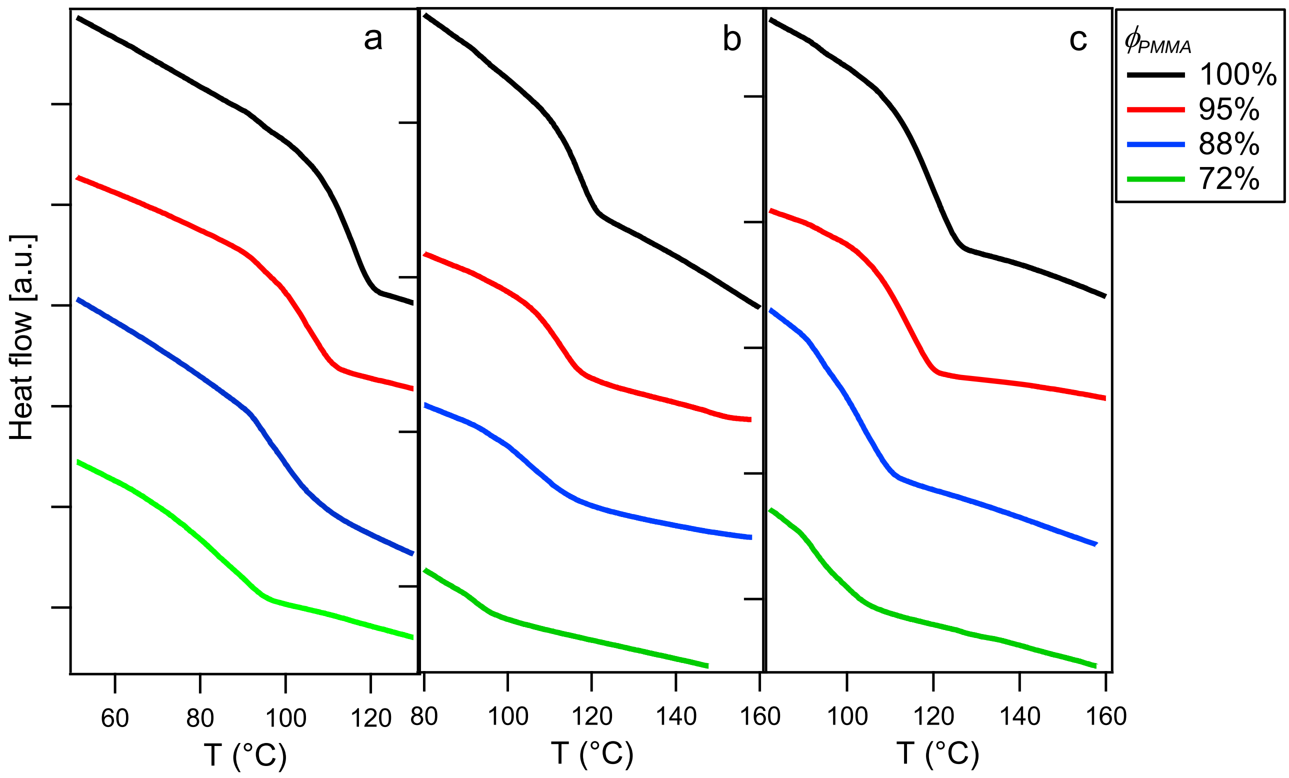

Supplementary figure 5. DSC measurements with varying ϕ_{PMMA} at scan rate of 10K min^{-1} for **a.** SiO_2-PEG_{2k}/$PMMA_{55k}$ **b.** SiO_2-PEG_{2k}/$PMMA_{165k}$ and **c.** SiO_2-PEG_{2k}/$PMMA_{280k}$. Curves have been displaced vertically to avoid crowding in the plot.

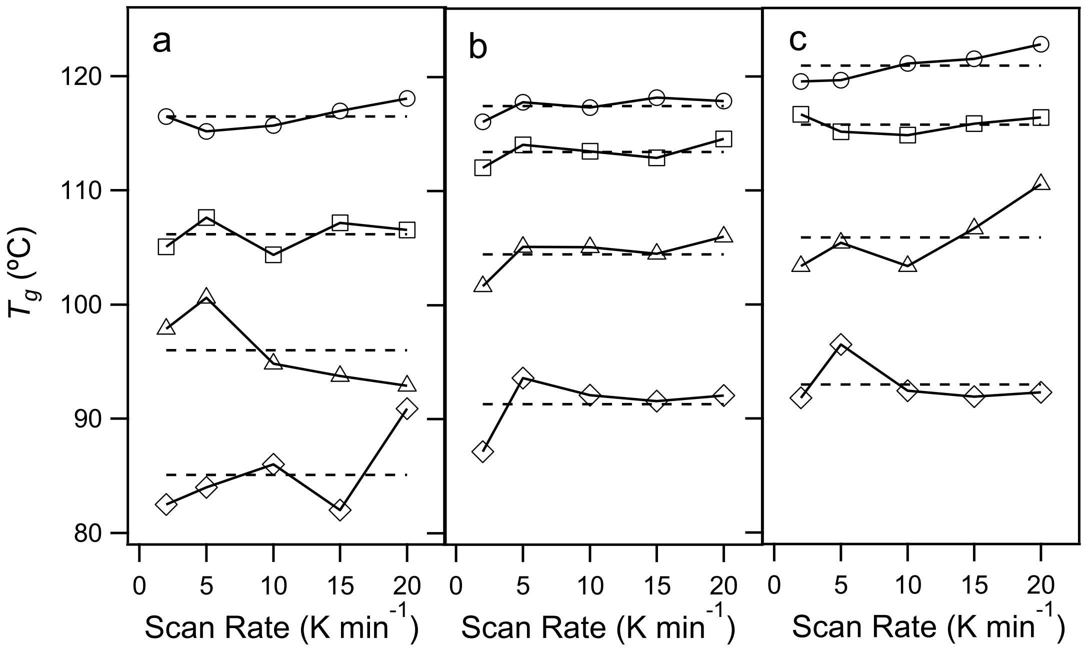

Supplementary figure 6. DSC scan rate dependence of T_g values for **a**, SiO_2-PEG_{2k}/$PMMA_{55k}$ **b**, SiO_2-PEG_{2k}/$PMMA_{165k}$ and **c**, SiO_2-PEG_{2k}/$PMMA_{280k.}$ ϕ_{PMMA} = 100% (circles), ϕ_{PMMA} = 95.2%, (squares) ϕ_{PMMA} = 88%(triangles) and ϕ_{PMMA} = 72% (diamonds). Dashed lines represent the mean T_g value. Results indicate that the T_g values obtained are independent of the scan rate. Error bar smaller than symbol size are not shown.

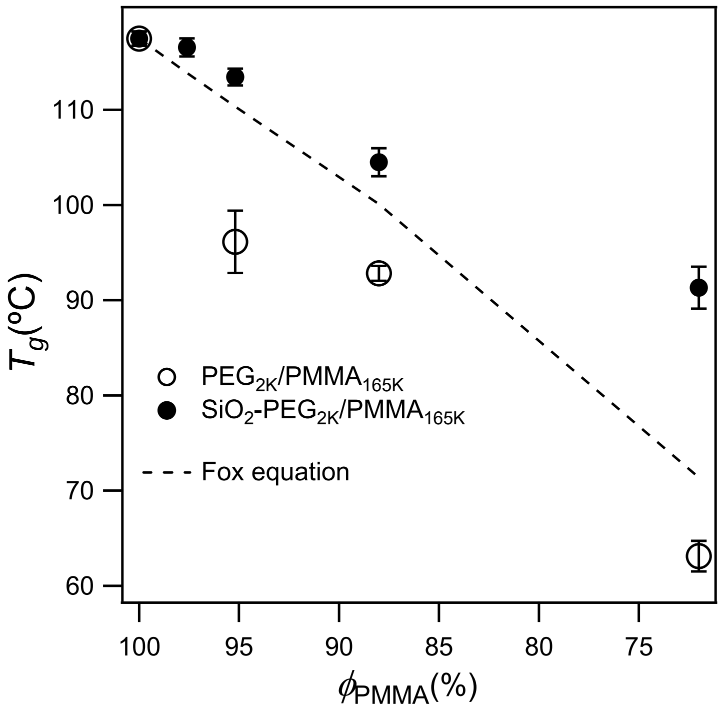

Supplementary figure 7. T_g values obtained for SiO_2-PEG_{2k}/$PMMA_{165k}$ obtained from DSC measurements. Evidently both tethered and free PEG chains reduce the T_g values of the PNC and particle free blend respectively, with decreasing PMMA content. A negative deviation in T_g values of the PEG/PMMA blends as compared to the values predicted by the simple mixing rule (Fox relation $T_g = \frac{m_{PEG}T_g + m_{PMMA}T_g}{m_{PEG} + m_{PMMA}}$) was observed due to PEG/PMMA miscibility. Error bars represent one standard deviation.

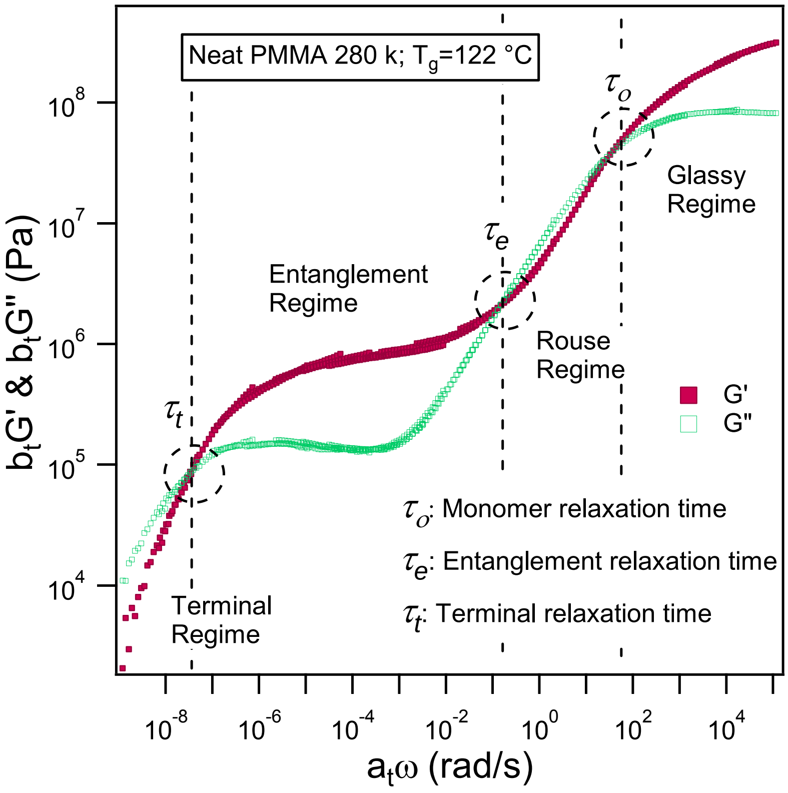

Supplementary figure 8 . Characteristic TTS master curve for neat PMMA 280k representing different host relaxation regimes. Relaxation times (τ_o, τ_e, τ_t) can be extracted from the cross over between the G' and G" as shown. Error bars smaller than symbol size are not shown.

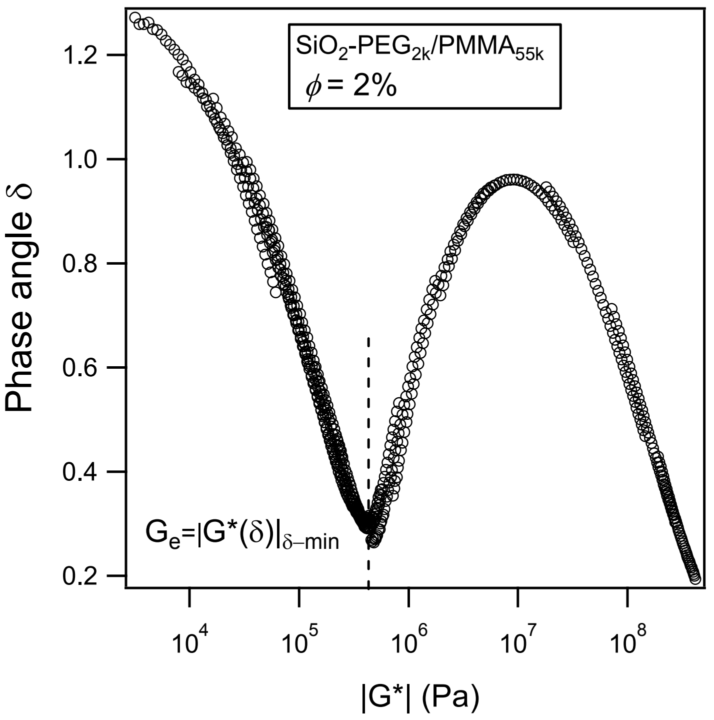

Supplementary figure 9. Representative Phase angle δ vs $|G^*|$ plot for SiO_2-PEG_{2k}/$PMMA_{55k}$ ϕ=2%. The minima in the plot as shown was used to obtain the G_e value using Van Gurp[46] analysis. Error bars smaller than symbol size are not shown.

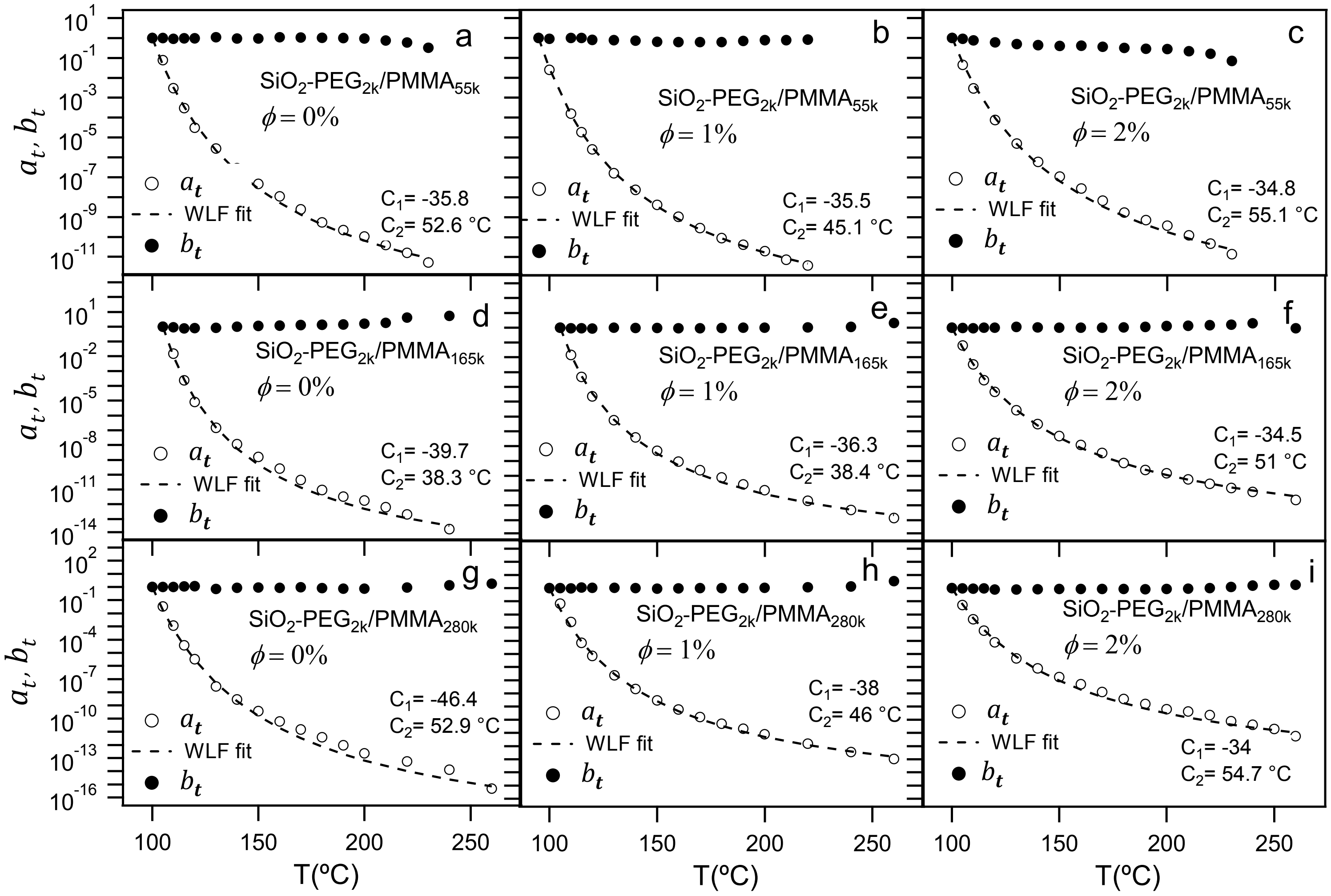

Supplementary figure 10. Shift factors employed in obtaining TTS master curves. **a,b,c** for SiO_2-PEG_{2k}/$PMMA_{55k}$ ϕ= 0%,1% and 2% respectively. **d,e,f** for SiO_2-PEG_{2k}/$PMMA_{165k}$ ϕ=0%,1% and 2% respectively and **g,h,i** for SiO_2-PEG_{2k}/$PMMA_{280k}$ ϕ =0%,1% and 2% respectively. Dotted line is the WLF fit $\log(a_t) = \frac{-C_1(T - T_r)}{C_2 + (T - T_r)}$ with C_1 and C_2 mentioned in the plots.

System	$B_{2,PNC}$ / $B_{2,HS}$
SiO_2-PEG_{2k}/$PMMA_{55k}$	3.2
SiO_2-PEG_{2k}/$PMMA_{165k}$	6.4
SiO_2-PEG_{2k}/$PMMA_{280k}$	5.3

Supplementary table 1. Second Virial coefficient obtained for PNCs from SAXS measurements in comparison with hard sphere values.
